# Stomatal Conductance Measurement for Toxicity Assessment in Zero-Effluent Constructed Wetlands: Effects of Landfill Leachate on Hydrophytes

**DOI:** 10.3390/ijerph16030468

**Published:** 2019-02-05

**Authors:** Andrzej Białowiec, Jacek A. Koziel, Piotr Manczarski

**Affiliations:** 1 Faculty of Life Sciences and Technology, Wroclaw University of Environmental and Life Sciences, 37a Chełmońskiego Str., 51-630 Wrocław, Poland; 2 Department of Agricultural and Biosystems Engineering, Iowa State University, Ames, IA 50011, USA; koziel@iastate.edu; 3 Department of Environmental Engineering, Faculty of Building Services, Hydro and Environmental Engineering, Warsaw University of Technology, 00-653 Warsaw, Poland; piotr.manczarski@pw.edu.pl

**Keywords:** environmental pollution, remediation, landfill leachate, constructed wetlands, hydrophytes, leaf stomatal conductance, plants transpiration, LOEC, environmental analysis, environmental assessment

## Abstract

In this research, we explore for the first time the use of leaf stomatal conductance (g_s_) for phytotoxicity assessment. Plants respond to stress by regulating transpiration. Transpiration can be correlated with stomatal conductance when the water vapor pressure gradient for transpiration is constant. Thus, our working hypothesis was that the g_s_ measurement could be a useful indicator of the effect of toxic compounds on plants. This lab-scale study aimed to test the measurement of g_s_ as a phytotoxicity indicator. Our model plants were two common hydrophytes used in zero-effluent constructed wetlands for treating landfill leachate. The toxic influence of two types of leachate from old landfills (L1, L2) on common reed (*Phragmites australis* (Cav.) Trin. ex Steud.) and sweet flag (*Acorus calamus* L.) was tested. The g_s_ measurements correlated well with plant response to treatments with six solutions (0 to 100%) of landfill leachate. Sweet flag showed higher tolerance to leachate solutions compared to common reed. The estimated lowest effective concentration (LOEC) causing the toxic effect values for these leachates were 3.94% of L1 and 5.76% of L2 in the case of reed, and 8.51% of L1 and 10.44% of L2 in the case of sweet flag. Leachate L1 was more toxic than L2. The leaf stomatal conductance measurement can be conducted in vivo and in the field. The proposed approach provides a useful parameter for indicating plant responses to the presence of toxic factors in the environment.

## 1. Introduction

Policy plans are being made to phase out the present day landfilling of municipal solid waste (MSW) with technologies consistent with the zero-waste, waste-to-carbon [1], and circular economy goals. However, even with the advances of the circular economy, the leachate of old, legacy landfills will need to be managed. One of the promising technologies for landfill leachate treatment and disposal is because of a considerable decrease in leachate volume due to evapotranspiration from zero-effluent constructed wetlands (CWs) [2,3,4,5,6,7]. Plants have a critical role in determining the dynamics of water loss, mainly by controlling the rate of water loss through evaporation and plant transpiration, i.e., evapotranspiration (ET). The ET of emergent hydrophytes (aquatic plants) in CWs is significant, reaching levels that are 7~8 times higher than actual evaporation without plants [7].

The leachate ET technology in zero-effluent CWs relies on evaporation from free water and soil surfaces and plant transpiration. An important CW design consideration is the toxic properties of leachate and the nutrients for improved plant growth conditions. With these eco-technologies for landfill leachate treatment, plants—typically hydrophytes—are exposed to leachate containing both nutrients and toxic compounds, e.g., ammonium and heavy metals. Hydrophyte response differs with soil and climatic conditions, which, in turn, influence the treatment efficiency related to ET. As ET is the main mechanism of leachate treatment, this parameter may also be used as an indicator of plant activity [8,9].

In this research, we explore for the first time the use of leaf stomatal conductance (*g_s_*), which is correlated to plant transpiration, to determine the toxic influence of harmful compounds in landfill leachate on plants. The advantages of the proposed measurement of *g_s_* as a toxicity indicator are that it is (1) less invasive to plants, (2) an easily measurable parameter, and (3) a measurement applicable to the on-site, field scale assessment of hydrophytes in CWs [10] and plants in general.

Therefore, we hypothesized that the direct measurement of leaf stomatal conductance might indicate a plant’s response to toxic substances in the environment and that the stomatal conductance will decrease with the increase of toxicant concentration. The proposed new method allows observation of the effect of toxicants on plant activity without damaging the plants in vivo.

The aim of this initial lab-scale study was to determine the influence of potentially toxic substances in two types of landfill leachate on leaf stomatal conductance of two hydrophyte species and to present an innovative approach to using the measurement of leaf stomatal conductance as a phytotoxicity indicator.

## 2. Materials and Methods

### 2.1. Landfill Leachate and Hydrophytes

Landfill leachate originating from two MSW landfills were used in the experiment: (L1) from Zakurzewo, near Grudziadz, Poland; and (L2) for Wola Pawłowska, near Ciechanów, Poland. The detailed multicomponent and initial chemical and physical properties of the landfill leachates used in the experiment have been presented by Białowiec [9]. Two emergent hydrophyte species were used: common reed (*Phragmites australis* (Cav.) Trin. ex Steud and sweet flag (*Acorus calamus* L.). The average plant weight and length was 19.5 ± 11.6 g and 42.1 ± 11.2 cm, and 34.2 ± 16.9 g and 66.2 ± 10.8 cm for *P. australis* and *A. calamus*, respectively.

### 2.2. The Experimental Matrix

The experiment was designed on two groupings of landfill leachate (L1, L2) and two plant species (*P. australis*, *A. calamus*). The independent variable was the concentration of landfill leachate in the solution; i.e., 0% (tap water), 6.25%, 12.5%, 25%, 50%, and 100%. Each variant of the experiment was repeated n = 5 times. The experimental matrix with 24 variants is shown in Table 1. The measured leaf stomatal conductance (g_s_) was used as the dependent variable.

The plants were cultivated in 1.5 L bottles placed in boxes. Five bottles representing the same variant (replicates) were placed in one box, so each box represented one specified variant. The boxes were arranged in a greenhouse along the window and in accordance with experimental factors: *P. australis*—L1 0%/100%, L2 0%/100%; *A. calamus*—L1 0%/100%, L2 0%/100%; i.e., a total of 120 bottles arranged in 24 boxes (variants) with 5 replicates per box. To avoid any potential mistakes during measurements and data recording, the individual plants’ spatial arrangement was not randomized. The greenhouse window length was 25 m, which ensured that all plants had similar lighting conditions. The experiment was conducted at 25.4 ± 6.1 °C. The greenhouse location was 53’74” N, and 20’44” E.

### 2.3. Experimental Procedure

The experimental procedures were similar to those described by Białowiec et al. [11]. Briefly, the plants were pre-cultivated in tap water for 4 weeks. After that, the 4-week-old plants were placed in bottles with solutions of landfill leachate (Table 2). The minimum 4-week duration of the plants’ hydroponic exposure to leachate was used according to the previous experiments that were focused on the influence of landfill leachate on transpiration rate, which was measured gravimetrically [8,9]. Gravimetric measurements, while accurate, have a low potential to be used in field work. The leaf stomatal conductance measurement we advanced here is suited well for on-site field work. The water was topped up in each bottle weekly to the ¾ (1.125 L) level. Only tap water (i.e., without leachate) was used to replenish water losses due to transpiration in both control and treatment.

The leaf stomatal conductance (*g_s_*) was measured daily in week 4, i.e., after 3 weeks of leachate treatment, using an AP-4-UM-3 porometer (Delta-T Devices, Cambridge, UK). The porometer was calibrated before measurements using the direct calibration technique recommended by the manufacturer. The porometer was equipped with a molded polypropylene calibration plate with six groups of holes; the rate of diffusion of water vapor through these holes has been carefully verified. Water vapor was provided by backing the plate with dampened paper. The sensor head was clipped onto the calibration plate, and readings were stored from each of the six standard calibration positions. Calibrations and measurements followed a detailed description in the user manual [12].

The leaf stomatal conductance was measured for each plant. Five randomly selected locations on leaves (large enough to allow the proper placement of the porometer sensor) were selected each day. A total of 4200 measurements of g_s_ were made, taking into consideration the 5 repetitions of the variant (box), 5 measurement locations on each plant, 2 plant species, 2 leachate typed, 6 leachate concentrations, and 7 days. The obtained data were incorporated into the subsequent statistical analyses as unweighted means for each plant, leachate type, and leachate dilution.

### 2.4. Toxicity Evaluation—the Lowest Effective Concentration causing a Toxic Effect (LOEC)

The inhibiting effect (*I*) caused by landfill leachate solutions was calculated with:

(1) $$I=100\frac{\left(C-T\right)}{C}$$

where:

*I* = effect caused by each landfill leachate solution (%);
*C* = mean measured leaf stomatal conductance for control treated with tap water only (mmol H_2_O·m^−2^·s^−1^);
*T* = mean measured leaf stomatal conductance for plants treated with landfill leachate solutions (mmol H_2_O·m^−2^·s^−1^).

The estimated *I* were then examined in relation to leachate concentrations, and LOEC values were estimated using the following function:

(2) $$I=a_{1}-e^{\left(LOEC-a_{2}\cdot C_{r}\right)}$$

where:

*C_r_* = landfill leachate solution concentration used in the experiment (%);
*a*_1_ (%) and *a*_2_ (−) = best fit constants [13].

The LOEC was therefore estimated through knowing the inhibition for a given leachate concentration and knowing that *a*_1_ > *I* and 0 < *a*_2_ < 1 from the transformed Equation (2):

(3) $$LOEC=ln\left(a_{1}-I\right)+a_{2}\cdot C_{r}$$

The best fit constants *a*_1_, and *a*_2_ were determined experimentally.

### 2.5. Statistical Procedures

The analysis of variance between mean values of estimated leaf stomatal conductance (g_s_) of *P. australis*—L1, *P. australis*—L2, *A. calamus*—L1, and *A. calamus*—L2 groups to indicate the influence of leachate concentration (*Cr*) was carried out with the one-way ANOVA test (followed by post–hoc Tukey’s test) at a significance level of *p* < 0.05. The estimation of LOEC values was completed with non-linear regression and the calculation of the determination coefficient R^2^. The Statistica 12.0 software package (Statsoft Polska, Kraków, Poland) was used.

## 3. Results

**Figure 1:**
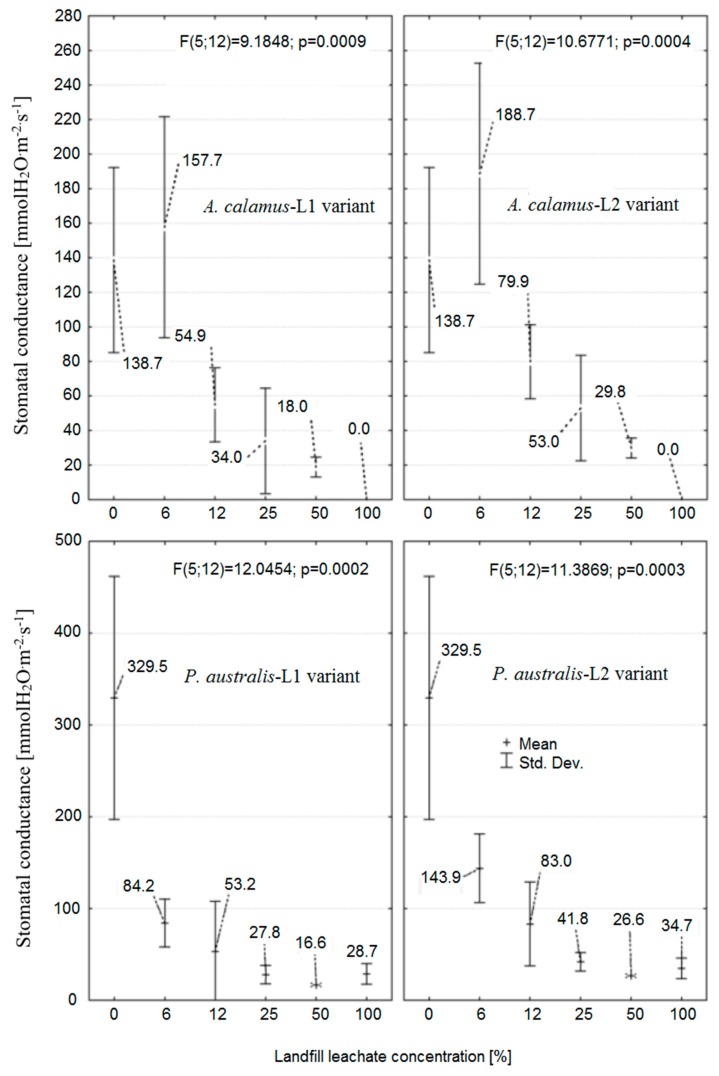
Measured mean (± SD (standard deviation)) leaf stomatal conductance of sweet flag (*Acorus calamus* L.) in the upper part and reed (*Phragmites australis* (Cav.) Trin. ex Steud) in the lower part as a function of leachate concentration treatment. Plants were cultivated for 4 weeks in landfill leachate solutions L1 and L2 at six landfill leachate concentrations from 0 (control) to 100 %. The F-Snedecor statistic (F) and probability (*p*) indicating the significance of the difference between mean values are given.

The measurements of *g_s_* confirmed the usefulness of this method for the observation of plant responses to toxicants in the environment. Plants responded by reducing the *g_s_* expressed in mmol H_2_O·m^−2^·s^−1^ (Figure 1). In the case of reed, plants growing in the solution with an L1 concentration of 6.25% reduced *g_s_* by about 74% compared to tap water (Figure 1). Plants submerged in 6.25% solution of L2 reduced *g_s_* by about 56% in relation to control. Further increase of leachate content had no statistically significant influence on the measured changes in *gs*. Reed response measured as *g_s_* was greater in the case of L1 than L2.

The measured leaf stomatal conductance was also capable of differentiating the response of sweet flag and reed for the same leachate type and concentration. The lower leachate content in the solution (6.25%) had a positive and significant (*p* < 0.05) influence on the *g_s_* value (Figure 1) mostly due to the fertilization effect of nutrients present in the leachate under a relatively low content of toxicants. The further increase of leachate content caused successive decreases of *g_s_* until complete inhibition in the case of the raw leachate (100%).

The plant’s transpiration rate is correlated to leaf stomatal conductance. Therefore, the hypothesis that direct measurement of g_s_ may indicate a plant’s response to toxic factors was confirmed in this controlled environment. To better understand the observed phenomenon of toxic compound influence on a plants’ stomatal conductance, more research should be done, including the influence of the lighting, the age of the plant, leaf development [14,15], the plant physiological status and particularly plant water status, indicators of saline, and osmotic stress [16]. Additionally, more work should be done on any potential chemical transformation such as ammonification, nitrification, organics degradation, a plants’ uptake in relation to a possible plant’s survival mechanisms under exposure to a mixture of complex toxicants, and selected physiological interactions between root zone and leaf physiology.

**Figure 2:**
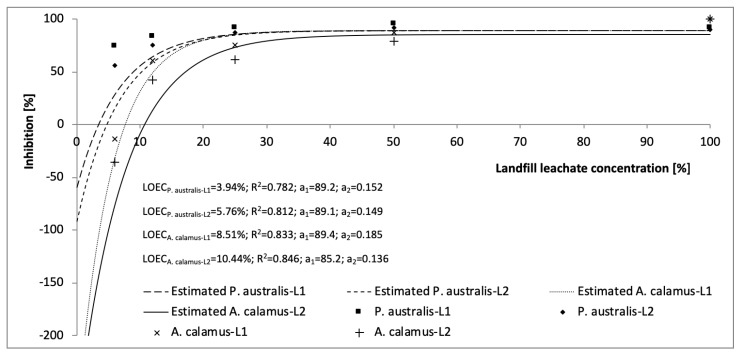
The estimation of lowest effective concentration causing the toxic effect (lowest effective concentration [LOEC]) values of landfill leachate L1 and L2, on leaf stomatal conductance of *P. australis*, and *A. calamus*; the a_1_ (%) and a_2_ (−) = best-fit constants, R^2^ = determination coefficients.

The LOEC values of leachate L1 and L2 were estimated for both plant species. The coefficient of determination value was relatively high in the range of ~0.782–0.846 (Figure 2). L1 toxicity was higher than that of L2 (lower values of LOEC). LOEC values were higher in sweet flag than reed for both types of leachate. Reed LOEC values were between 3.94 and 5.76% (Figure 1). Sweet flag values varied from 8.51 to 10.44%, showing higher resistance than reed.

## 4. Conclusions

The leaf stomatal conductance measurements were correlated with plant response to toxics in landfill leachate. Sweet flag was more resistant to landfill leachate treatment than reed. Based on these initial lab-scale studies, it is recommended that the leachate effluent content discharged into the zero-effluent constructed wetland system should not exceed ~8–11% and ~3–6% for sweet flag and reed, respectively, especially during the initial growth stage. The recommended landfill leachate dose rate could be adjusted to the local precipitation regime. Further research should be continued on the development and benchmarking of this method compared to other protocols. The pros of the proposed leaf stomatal conductance measurement warrant further research to develop it into a robust and minimally-invasive approach for measuring plant response to the presence of toxins in the environment.

## Figures and Tables

**Table 1:** Experimental design matrix variants.

Plant Species	Leachate Type	Leachate Concentration (%)					
*P. australis*	L1	0 (tap water)	6.25	12.5	25.0	50.0	100.0
	L2						
*A. calamus*	L1						
	L2						

**Table 2:** The timeline of the experiment.

Timeline of the Experiment, Weeks							
1	2	3	4	5	6	7	8
Pre-cultivation of plants in tap water				Treatment of plants with leachate solutions			
							(g_s_) *

* daily measurements of leaf stomatal conductance (*g_s_*) during week 8.
